# In vitro analysis of the effects of intronic variants c.940+3_940+6del, c.941-3C>G, and c.2389+5G>A in the LDLR gene on pre-mRNA splicing using a minigene assay

**DOI:** 10.18699/vjgb-26-81

**Published:** 2026-09

**Authors:** V.Yu. Danilchenko, D.E. Ivanoshchuk, O.V. Timoshchenko, E.A. Panina, P.S. Orlov, E.V. Shakhtshneider

**Affiliations:** Institute of Cytology and Genetics of the Siberian Branch of the Russian Academy of Sciences, Novosibirsk, Russia; Institute of Cytology and Genetics of the Siberian Branch of the Russian Academy of Sciences, Novosibirsk, Russia; Institute of Internal and Preventive Medicine – Branch of the Institute of Cytology and Genetics of the Siberian Branch of the Russian Academy of Sciences, Novosibirsk, Russia; Institute of Cytology and Genetics of the Siberian Branch of the Russian Academy of Sciences, Novosibirsk, Russia; Institute of Cytology and Genetics of the Siberian Branch of the Russian Academy of Sciences, Novosibirsk, Russia; Institute of Cytology and Genetics of the Siberian Branch of the Russian Academy of Sciences, Novosibirsk, Russia

**Keywords:** familial hypercholesterolemia, LDLR gene, intronic variants, minigenes, functional analysis, NGS, семейная гиперхолестеринемия, ген LDLR, интронные варианты, минигены, функциональный анализ, NGS

## Abstract

Familial hypercholesterolemia (FH) is an autosomal codominant disorder characterized by impaired clearance of low-density lipoproteins from the bloodstream, markedly elevated plasma total cholesterol and low density lipoprotein cholesterol levels, and early onset of atherosclerosis and cardiovascular disease. FH is one of the most common monogenic disorders in humans. The majority of FH cases are caused by pathogenic variants in three genes: LDLR (OMIM: 143890), APOB (OMIM: 107730), and PCSK9 (OMIM: 607786). More than 80 % of FH cases are associated with mutations in the LDLR gene, located on chromosome 19. In 25–75 % of patients with a clinical FH phenotype, no pathogenic variant is identified in these genes. Whole-exome sequencing and targeted gene panel sequencing of the LDLR, APOB, PCSK9, and LDLRAP1 genes were performed in patients with an FH phenotype, followed by confirmation of the identified variants by Sanger sequencing. Three unrelated probands were found to carry intronic LDLR variants for which functional evidence was previously unavailable. The aim of this study was to functionally assess in vitro the effects of three intronic LDLR variants (NM_000527.5: c.940+3_940+6del, c.941-3C>G, and c.2389+5G>A) on pre-mRNA splicing using a minigene assay. Minigene constructs encompassing target exons with flanking intronic sequences were generated and transfected into the HEK293 and HeLa cell lines. The deleterious effect of all three variants on pre-mRNA splicing was confirmed. The four-nucleotide deletion c.940+3_940+6del resulted in two aberrant transcripts: inclusion of six nucleotides from intron 6 and complete retention of the minigene intronic sequence. The c.941-3C>G variant caused loss of the canonical acceptor splice site and activation of a cryptic site, with inclusion of intronic nucleotides from intron 6. The c.2389+5G>A variant resulted in exon 16 skipping. Functional in vitro analysis is a key tool for the molecular verification of intronic variants of uncertain clinical significance and, in conjunction with clinical data, supports the establishment of a definitive molecular diagnosis.

## Abstract (ru)

Семейная гиперхолестеринемия (СГХС) – аутосомно-кодоминантное заболевание, характеризующееся нарушением выведения липопротеинов низкой плотности из кровотока, выраженным повышением уровня общего холестерина и холестерина липопротеинов низкой плотности в крови и ранним развитием атеросклероза и сердечно-сосудистых заболеваний. СГХС является наиболее распространенным моногенным заболеванием у человека. Самые частые причины развития заболевания – мутации в трех генах: LDLR (OMIM: 143890), APOB (OMIM: 107730) и PCSK9 (OMIM: 607786). Более 80 % случаев СГХС обусловлено патогенными вариантами гена LDLR, локализованного на хромосоме 19. У 25–75 % пациентов с фенотипом СГХС патогенный вариант в указанных генах не обнаруживается. Полноэкзомное и таргетное секвенирование генов LDLR, APOB, PCSK9, LDLRAP1 было проведено у пациентов с фенотипом СГХС с последующим подтверждением выявленных вариантов секвенированием по методу Сэнгера. У трех неродственных пробандов обнаружены интронные варианты гена LDLR, для которых ранее не проводилась функциональная верификация. Целью работы стала функциональная оценка in vitro влияния трех интронных вариантов гена LDLR (NM_000527.5: c.940+3_940+6del, c.941-3C>G, c.2389+5G>A) на процесс сплайсинга с использованием генетических конструкций – минигенов. Для функциональной верификации in vitro были созданы минигены, включающие исследуемые экзоны с фланкирующими интронными областями. Анализ сплайсинга проводили в клеточных линиях HEK293 и HeLa. В результате анализа подтвержден патогенный эффект всех трех выявленных вариантов на сплайсинг пре-мРНК. Делеция четырех нуклеотидов c.940+3_940+6del приводила к образованию двух аберрантных транскриптов – включению шести нуклеотидов интрона 6, а также к полному удержанию интронной последовательности минигена. Вариант c.941-3C>G обусловливал потерю акцепторного сайта сплайсинга и активацию криптического, с включением нуклеотидов интрона 6. Замена c.2389+5G>A приводила к пропуску экзона 16. Функциональный анализ in vitro – ключевой инструмент молекулярной верификации интронных вариантов с неопределенной клинической значимостью и в совокупности с клиническими данными помогает в установлении окончательного диагноза.

## Introduction

Familial hypercholesterolemia (FH) is an inherited disorder characterized by impaired clearance of low-density lipoprotein (LDL) particles from the circulation, markedly elevated plasma total cholesterol and low density lipoprotein cholesterol levels, and premature atherosclerosis and cardiovascular disease (Austin et al., 2004; Nordestgaard et al., 2013). FH is a monogenic disorder with a predominantly autosomal codominant pattern of inheritance, whereby the number of pathogenic alleles determines the severity of clinical manifestations (Freiberger, 2026).

The prevalence of heterozygous FH in the general population is approximately one in 311 individuals, making it one of the most common inherited disorders in humans (Hu et al., 2020). Sustained hypercholesterolemia leads to premature atherosclerosis and early-onset cardiovascular complications. Timely molecular genetic diagnosis confirms the clinical diagnosis and guides the selection of optimal lipid-lowering therapy, thereby contributing to reduced mortality from atherosclerotic disease (Ahmad et al., 2026).

FH is most commonly caused by pathogenic variants in one of three genes: LDLR (low-density lipoprotein receptor, OMIM: 143890), APOB (apolipoprotein B, OMIM: 107730), and PCSK9 (proprotein convertase subtilisin/kexin type 9, OMIM: 607786). Variants in LDLR account for 85–90 % of molecularly confirmed FH cases, and more than 3,000 pathogenic variants in this gene have been reported to date in patients with lipid metabolism disorders (Ahmad et al., 2026). The LDLR gene (19p13.2) includes 18 exons and encodes the LDL receptor protein comprised of 860 amino acids (Hobbs et al., 1990). The primary function of the LDLR protein is to mediate the clearance of LDL particles from the circulation via binding to apolipoprotein B-100, thereby promoting their lysosomal degradation and maintaining cellular and plasma cholesterol homeostasis (Brown, Goldstein, 1979; Südhof et al., 1985; Segrest et al., 2001).

In 25–75 % of patients with a clinical FH phenotype, no pathogenic variant is identified in the genes mentioned above (Freiberger, 2026). One contributing factor is the difficulty in clinical interpretation of variants located outside the coding sequences and canonical splice sites. Substitutions at donor site positions +1/+2 and acceptor site positions −1/−2 disrupt pre-mRNA splicing and are generally straightforward to interpret. By contrast, splice region variants and synonymous substitutions can impair spliceosomal recognition of donor and acceptor sites to varying degrees, activate cryptic splice sites, cause exon skipping or intron retention, and ultimately give rise to aberrant transcripts and loss of protein function (Abramowicz, Gos, 2018). In silico predictions for such variants are often contradictory, and in the absence of functional evidence, most are classified as variants of uncertain significance (VUS) (Walker et al., 2023). Variants of this type have been reported in LDLR in patients with FH (Kulseth et al., 2010; Reeskamp et al., 2018, 2021), however, their functional verification remains uncommon.

A widely used experimental approach for assessing the impact of intronic variants on splicing in multi-exon genes is the minigene assay. This method can be used when patientderived RNA is unavailable, as it enables the evaluation of splicing outcomes in a cell-based in vitro system (Abramowicz, Gos, 2018).

The aim of this study was to functionally evaluate the effects of three intronic LDLR variants (c.940+3_940+6del, c.941-3C>G, and c.2389+5G>A) identified in patients with a clinical FH phenotype on pre-mRNA splicing, using an in vitro minigene assay.

## Materials and methods

Patients with familial hypercholesterolemia enrolled in this study were examined at the Clinical Diagnostic Department of the Research Institute of Internal and Preventive Medicine – Branch of the Institute of Cytology and Genetics, Siberian Branch of the Russian Academy of Sciences (IIPM – Branch of ICG SB RAS). The study protocol was approved by the Local Ethics Committee of IIPM – Branch of ICG SB RAS (Protocol No. 68, June 4, 2019). Written informed consent was obtained from each participant, or from a parent or legal guardian on behalf of minor participants, prior to enrollment.

The clinical diagnosis of FH was established using the Dutch Lipid Clinic Network (DLCN) diagnostic criteria (Table 1) and the Simon Broome Register criteria (Table 2). The DLCN scoring system classifies patients into four probability categories for FH diagnosis based on cumulative point scores (Tables 1 and 2) (Ezhov et al., 2019).

**Table 1.**
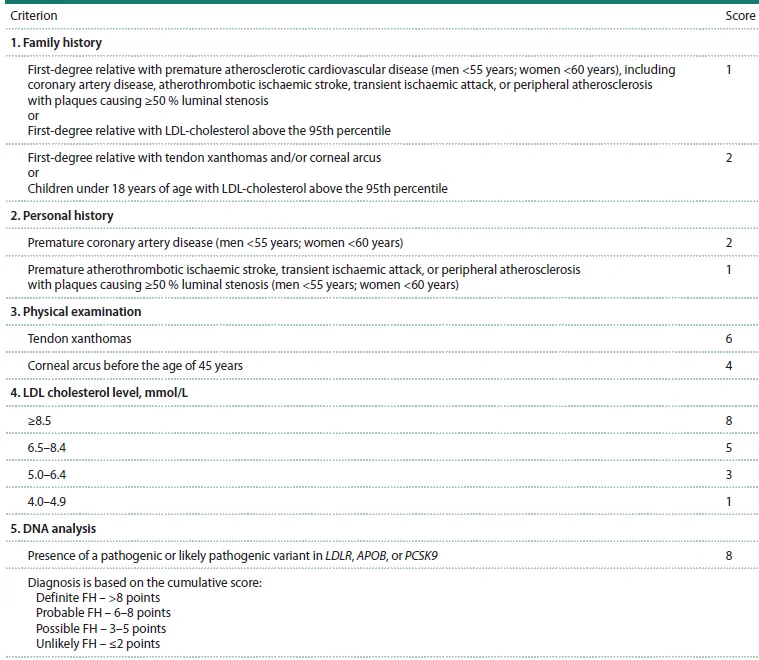
DLCN diagnostic criteria for the diagnosis of heterozygous FH in adults (18 years of age and older)

**Table 2.**
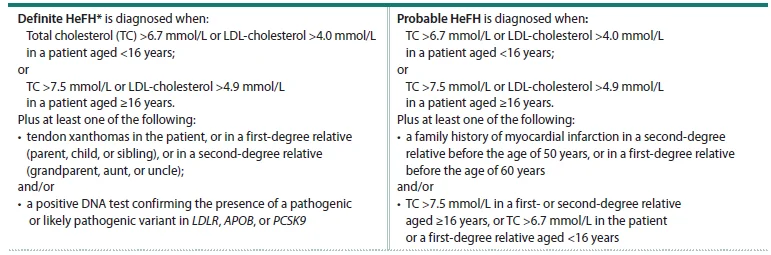
Simon Broome Register diagnostic criteria for heterozygous familial hypercholesterolemia * HeFH – heterozygous familial hypercholesterolemia.

The diagnosis is established based on the sum of points obtained across all five categories. Within each category, scores are not cumulative – only a single criterion yielding the highest score is counted.

All study participants underwent clinical examination, vascular ultrasound imaging, and venous blood sampling for biochemical analysis, including total cholesterol (TC), LDL cholesterol (LDL-C), HDL cholesterol (HDL-C), triglycerides (TG), atherogenic index (AI), and fasting plasma glucose. Molecular genetic analysis was performed in all participants

Genomic DNA for subsequent analysis was extracted from peripheral venous blood using the phenol–chloroform extraction method (Sambrook, Russell, 2006).

Whole-exome sequencing was performed on the FASTA Seq 300 platform (GeneMind Biosciences, China). Library preparation was carried out using the SG GM Ultra-96 kit (cat. No. #SG_GM_plus-96, Raissol Bio, Russia). Exome capture was performed using the VAHTS Target Capture Core Exome Panel (cat. No. NC001-01, Vazyme, China) according to the manufacturer’s protocol. The capture panel covered exonic sequences (~33.9 Mb) with limited coverage of adjacent intronic regions. Library quality was assessed on the Qsep1 platform (BiOptic, Taiwan), and library concentration was determined using a Qubit 2.0 fluorometer (Thermo Fisher Scientific, USA).

Raw sequencing data were processed using NGS Wizard software (Genomenal, version 2.11.0; https://genomenal.ru/). Alignment to the GRCh38 reference genome achieved a mapping rate of 99.99 %, with a properly paired alignment rate of 99.98 %; the duplication rate was 4.82 %. Mean per-nucleotide coverage was 129×. Nucleotide and exon numbering was based on the GenBank reference sequence NM_000527.4.

Variant filtering criteria were as follows: genotype quality ≥30, read depth ≥10, and minor allele frequency (MAF) in the gnomAD database (versions 3.2.1 and 4.1.1; https:// gnomad.broadinstitute.org) <0.01 %. Candidate variants were confirmed by Sanger sequencing using a GTZ G08 genetic analyzer (Gene Technologies of Health, Russia/China). For patient P03, variant detection was performed by targeted next-generation sequencing with hybridization capture-based library enrichment using a custom probe panel (Roche, Basel, Switzerland) according to the manufacturer’s recommended protocol. The panel was designed to cover all exons, introns, putative promoter regions, and 5ʹ-untranslated regions (5ʹUTR) of four genes: LDLR, APOB, PCSK9, and LDLRAP1. Sequencing was performed on the GS Junior platform (Roche, Basel, Switzerland).

The potential impact of the identified LDLR variants on pre-mRNA splicing was assessed by in silico analysis using the following prediction tools: Human Splicing Finder (https:// genomnis.com/hsf), SpliceAI (https://spliceailookup.broad institute.org/), and MaxEntScan (Shamsani et al., 2019).

The pathogenicity of the identified variants was evaluated in accordance with the ACMG/AMP variant classification guidelines (Richards et al., 2015) and the gene-specific rules of the ClinGen Familial Hypercholesterolemia Variant Curation Expert Panel (FH VCEP) (Chora et al., 2022).

Genomic fragments of the LDLR gene (NG_009060.1) containing exons and flanking intronic sequences were selected for minigene construction. The exon-trap vector pET01 (MoBiTec GmbH, Germany) was used in the study. Oligonucleotide primers for amplification of the relevant LDLR genomic fragments were designed using Primer3Plus (https://www. primer3plus.com/index.html) (Table 3). Genomic DNA from patients carrying variants c.940+3_940+6del, c.941-3C>G, and c.2389+5G>A served as PCR templates.

**Table 3.**
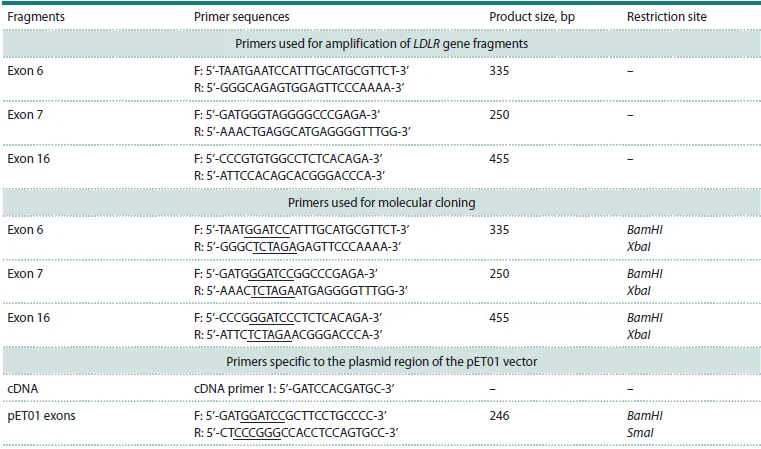
Primers used for amplification of LDLR gene fragments Notе. The 5’ ends containing recognition sites for restriction endonucleases are underlined.

Initial amplification was performed using the designed primers and Q5 High-Fidelity DNA Polymerase (New England Biolabs, USA), followed by re-amplification (re-PCR) with primers including recognition sites for the restriction endonucleases BamHI and XbaI at their 5′ ends (Table 3). PCR products and the pET01 vector were digested with BamHI and XbaI restriction endonucleases (New England Biolabs). The resulting fragments were cloned into the pET01 vector at the corresponding restriction sites using T4 DNA Ligase (SibEnzyme, Russia), and the constructs were transformed into Escherichia coli. Colony screening for minigene constructs carrying the studied variants and the wild-type sequence was performed by PCR with vector-specific primers (Table 3), agarose gel electrophoresis, and Sanger sequencing. Plasmid DNA was isolated using the Plasmid Midiprep kit (Evrogen, Russia). All minigenes were sequenced to confirm the absence of unintended sequence alterations.

Splicing analysis was initially performed using the HEK293 cell line and subsequently repeated using the HeLa cell line. Both cell lines were cultured in DMEM/F12 medium (Servicebio, China) supplemented with 10 % fetal bovine serum (Capricorn, Germany) and 2 mM penicillin/streptomycin (Capricorn) at 37 °C in a humidified incubator with 5 % CO2. Cell lines were transfected with either mutant or wild-type minigenes in 6-well plates (Eppendorf, Germany) using Lipofectamine 3000 (Thermo Fisher Scientific, USA) according to the manufacturer’s instructions.

Forty-eight hours post-transfection, total RNA was extracted from the cells using the RNA Solo kit (Evrogen). The isolated RNA was treated with RQ1 RNase-Free DNase (Promega, USA) to eliminate residual genomic DNA contamination. Complementary DNA (cDNA) was synthesized using the MMLV RT kit (Evrogen) with a primer specific to the plasmid region of the pET01 vector (Table 3). Minigene-derived transcripts were then amplified by PCR using the synthesized cDNA as template and primers specific to the exons of the pET01 vector (Table 3). PCR products were resolved by electrophoresis on a 3 % agarose gel. The nucleotide sequence of each amplified fragment was confirmed by Sanger sequencing.

Construction, editing, and annotation of nucleotide and protein sequences were performed using Unipro UGENE software, version 53.1 (Okonechnikov et al., 2012).

## Results

Molecular genetic analysis identified three LDLR variants potentially affecting pre-mRNA splicing in three unrelated probands presenting with a clinical FH phenotype (Table 4): c.940+3_940+6del, affecting the donor splice site of intron 6; c.941-3C>G, affecting the acceptor splice site of intron 6; and c.2389+5G>A, located within the donor splice site of intron 16. None of these variants had previously been functionally characterized in the published literature.

**Table 4.**
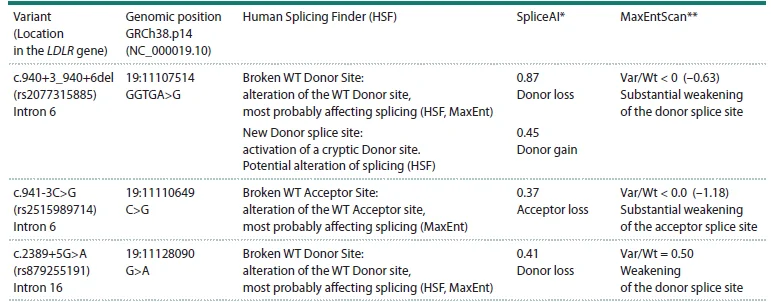
Results of in silico splicing analysis of LDLR variants * SpliceAI Δscores ≥0.80 are considered high-confidence predictions of splicing impact; ** the MaxEntScan Var/WT ratio <0.80 provides supportive evidence for pathogenicity, whereas the ratio ≥1.0 provides supportive evidence for a benign effect of the variant (Chora et al., 2022).

Proband P01 was a male born in 1989 (36 years of age at the time of examination) with marked hypercholesterolaemia: total cholesterol (TC) was 9.0 mmol/L and LDL cholesterol (LDLC) was 7.2 mmol/L. Triglycerides (TG) were 1.1 mmol/L and HDL cholesterol (HDL-C) was 1.3 mmol/L, consistent with the typical lipid profile of familial hypercholesterolaemia, characterized by pronounced elevation of TC and LDL-C (optimal values: TC <5.0 mmol/L, LDL-C <3.0 mmol/L in patients without additional risk factors) and normal TG levels (<1.7 mmol/L).

Family history was notable: his biological sister (38 years at the time of examination) had a markedly elevated TC level of 10.0 mmol/L. The father was also reported to have hypercholesterolaemia, but laboratory data were not available. The DLCN score for the proband was 7 points, corresponding to “probable FH” (6–8 points) (Ezhov et al., 2019). Molecular genetic testing identified the previously reported LDLR variant NM_000527.5:c.940+3_940+6del (GRCh38: chr19:11107514 GGTGA>G) in the proband. The proband’s father and sister were not tested for this variant as DNA samples were unavailable. The variant was absent from the general population in the gnomAD database and had previously been described in patients with familial hypercholesterolaemia (Rieck et al., 2020; Semenova et al., 2020; Miroshnikova et al., 2021; Nazarenko et al., 2023). According to ACMG/AMP criteria as specified by the ClinGen FH Variant Curation Expert Panel, c.940+3_940+6del was classified as a variant of uncertain significance (PM2, PP3, PP4).

The LDLR intronic variant NM_000527.5:c.941-3C>G (GRCh38: chr19:11110649 C>G) in intron 6 was identified in proband P02 and her mother. Proband P02 was a female born in 2001 (22 years of age at the time of examination); her maximum recorded TC was 7.8 mmol/L, and LDL-C, 5.5 mmol/L, exceeding the 95th percentile of reference values for women in this age group (Ezhov et al., 2019). Family history was strongly positive: her father had clinical coronary artery disease from the age of 45 years, experienced an acute myocardial infarction at age 50, and underwent coronary artery bypass grafting; her mother had TC levels up to 8 mmol/L and was receiving lipid-lowering therapy; her brother had marked hypercholesterolaemia with TC up to 10 mmol/L.

Ultrasound examination of the brachiocephalic arteries and arteries of the lower extremities revealed no atherosclerotic changes. The proband’s DLCN score was 4 points, corresponding to “possible FH” (3–5 points). The c.941-3C>G variant was absent from the general population in gnomAD. Other family members were unavailable for genetic testing. This variant had previously been reported in individuals with familial hypercholesterolaemia in Spain (Martín-Campos et al., 2018) and in Russia (Meshkov et al., 2021). Based on ACMG/AMP criteria as adapted by the ClinGen FH Variant Curation Expert Panel, c.941-3C>G was classified as a VUS (PM2, PP3, PP1_Supporting).

In proband P03, a 43-year-old male at the time of examination, we had previously identified a variant located at the donor splice site, NM_000527.5:c.2389+5G>A (GRCh38: chr19:11128090 G>A), in intron 16 of the LDLR gene (Shakhtshneider et al., 2017). From childhood, the proband had arterial hypertension, and in 2011 he suffered an acute myocardial infarction. Laboratory testing revealed markedly elevated lipid levels: TC – 11.5 mmol/L, LDL-C – 10.12 mmol/L, HDL-C – 0.89 mmol/L, and TG – 1.08 mmol/L. Family history was strongly positive for premature coronary artery disease: the father had an acute myocardial infarction at 45 years of age, and three paternal uncles had infarctions between 50 and 53 years of age. The proband’s older brother had hypercholesterolaemia and coronary artery disease and carried the same LDLR variant. The DLCN score was 17 points, corresponding to definite familial hypercholesterolaemia. Cascade screening identified the c.2389+5G>A variant in two of the proband’s three daughters (aged 7–8 years), both of whom had hypercholesterolaemia with LDL-C levels of 4.28–4.69 mmol/L. In the gnomAD population database (version 4.1.1), the variant is reported at a frequency of 0.0001239 %, with no homozygotes observed. The variant has previously been described in individuals with familial hypercholesterolaemia (Semenova et al., 2020). According to ACMG/AMP criteria as specified by the ClinGen FH Variant Curation Expert Panel, c.2389+5G>A was classified as a VUS (PM2, PP1_Moderate, PP3, PP4).

Based on in silico splicing predictions for the LDLR variants c.940+3_940+6del, c.941-3C>G, and c.2389+5G>A (Table 4), minigenes were constructed, each comprising a single exon and its flanking intronic regions (Fig. 1). For the minigene containing variant c.940+3_940+6del, the flanking intronic sequences were 116 and 96 bp in length, whereas for the c.941-3C>G construct, they were 51 and 79 bp. The shorter intronic segments in the latter construct reflect the fact that LDLR introns are composed predominantly of Alu repeat elements (~65 % of intronic sequence) (Amsellem et al., 2002).

**Fig. 1.**
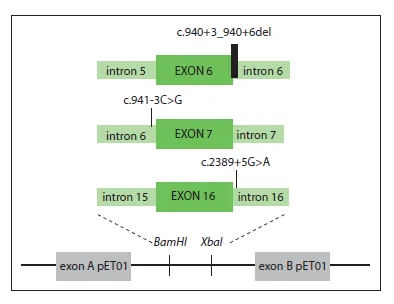
Scheme of minigenes with the c.940+3_940+6del, c.941-3C>G, and c.2389+5G>A LDLR variants. The pET01 fragments are colored in gray, the LDLR gene fragments, in green. BamHI and XbaI indicate the recognition sites for restriction endonucleases.

The c.940+3_940+6del variant is a 4-nucleotide deletion at positions +1 to +4 of intron 6; in silico analysis predicted loss of the canonical donor splice site with activation of a cryptic donor site (Table 4). In the wild type (WT) minigene, a single product of 369 bp was detected, corresponding to correct splicing with complete removal of introns and retention of exon 6. In contrast, analysis of the c.940+3_940+6del (MUT) minigene revealed two major products of 775 and 375 bp. The 775 bp fragment reflects loss of the donor splice site and inclusion of intron 6 of LDLR together with the intron of the pET01 vector in the mRNA. The 375 bp fragment represents loss of the canonical donor site and activation of a cryptic donor site, leading to inclusion of six nucleotides derived from intron 6 (Fig. 2a). These in vitro data demonstrate a pathogenic effect of c.940+3_940+6del, resulting in aberrant splicing.

**Fig. 2.**
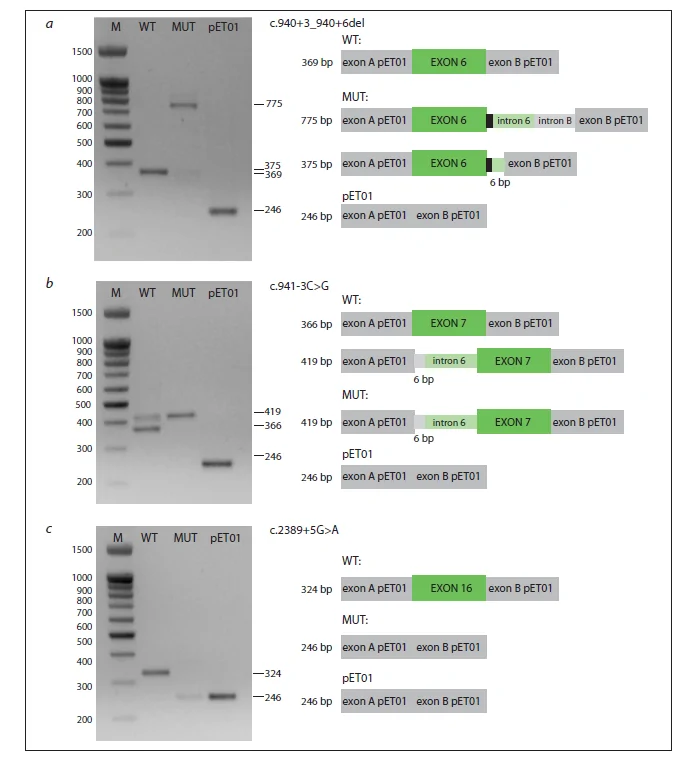
Results of minigene assay (obtained on the HEK293 cell line) for LDLR variants c.940+3_940+6del, c.941-3C>G, c.2389+5G>A and the corresponding wild types (WT). RT‑PCR products were visualized on 3 % agarose gels, and schematic representations of the observed splicing patterns are shown below each gel. The pET01 fragments are colored in gray, the LDLR gene fragments, in green. a – minigenes: (MUT) c.940+3_940+6del and wild type (WT); b – minigenes: (MUT) c.941-3C>G and wild type (WT); c – minigenes: (MUT) c.2389+5G>A and wild type (WT); pET01 – pET01 vector without insertion; M – DNA size marker (100 + 1,500 bp).

The c.941-3C>G variant is located at position −3 of intron 6 of LDLR, and in silico predictions for this variant were discordant (Table 4). In the wild-type minigene, two products were observed: a 366 bp product corresponding to correct splicing, and a 419 bp product containing a chimeric intron composed of six nucleotides from the pET01 intron and the entire intron 6 of LDLR (Fig. 2b). Retention of intron 6 in the wild-type minigene is likely related to specific features of the construct. In the minigene carrying the c.941-3C>G variant, a single fragment was detected, which also contained the same chimeric intron (six nucleotides from the pET01 intron and the full intron 6 of LDLR) (Fig. 2b). Thus, in vitro analysis confirmed that c.941-3C>G causes loss of the canonical acceptor splice site and activation of a cryptic splice site.

The c.2389+5G>A variant is located outside the canonical splice site at position +5 of intron 16. In vitro analysis confirmed the in silico prediction for c.2389+5G>A (Table 4), namely loss of the canonical donor splice site. In the minigene carrying this variant, the predominant product was 246 bp, corresponding in size to the empty pET01 vector (Fig. 2c). In vitro analysis confirmed the pathogenic effect of the c.2389+5G>A variant on splicing.

Considering the possible cell-type specificity of splicing regulation (Cooper, 2005), the experiment was repeated using the HeLa cell line. The results obtained in this cell line completely reproduced those obtained in HEK293 cells.

In vitro functional data were then incorporated into variant interpretation in accordance with ACMG/AMP pathogenicity criteria (Richards et al., 2015) and the gene-specific specifications of the ClinGen Familial Hypercholesterolemia Variant Curation Expert Panel (FH VCEP) (Chora et al., 2022). The minigene assay confirmed aberrant splicing for all three variants (c.940+3_940+6del, c.941-3C>G, and c.2389+5G>A), providing supportive functional evidence of pathogenicity (PS3_Supporting under FH VCEP specifications).

Variants c.940+3_940+6del and c.941-3C>G have been reported in patients with FH in several independent studies (Martín-Campos et al., 2018; Rieck et al., 2020; Semenova et al., 2020; Meshkov et al., 2021; Miroshnikova et al., 2021; Nazarenko et al., 2023). However, application of LDLRspecific FH VCEP criteria (Chora et al., 2022) indicates that the totality of accumulated evidence remains insufficient for reclassification to likely pathogenic. Accordingly, under the ACMG/AMP criteria, c.940+3_940+6del (PM2, PS3_ Supporting, PP4) and c.941-3C>G (PM2, PS3_Supporting, PP1_Supporting) remain classified as variants of uncertain significance.

In contrast, c.2389+5G>A was reclassified from a VUS to likely pathogenic, supported by criteria PM2, PS3_Supporting, PP1_Moderate, and PP4 under the ACMG/AMP framework and FH VCEP specifications.

## Discussion

In this study, we present an in vitro splicing analysis of three intronic LDLR variants – c.940+3_940+6del, c.941-3C>G, and c.2389+5G>A – using the exon-trap pET01 vector.

Canonical splice sites define exon–intron boundaries, are evolutionarily conserved, and are recognized by the spliceosome. Any variation in the sequence of these canonical sites may alter interactions between pre‑mRNA and spliceosomal subunits (Abramowicz, Gos, 2018). Recognition of the 5ʹ splice site by U1 snRNP occurs via base pairing between the 5ʹ end of U1 snRNA and pre‑mRNA over positions −3 to +6 relative to the exon–intron junction (Malard et al., 2022). Preservation of the invariant GT dinucleotide alone is therefore not a reliable predictor of donor site efficiency; a key determinant is the thermodynamics of U1 snRNA:5′ss duplex formation, in which the presence of a certain nucleotide at position +3 and further downstream makes a substantial contribution to duplex stability and donor site strength (Malard et al., 2022).

The c.940+3_940+6del variant results in deletion of four nucleotides at positions +1 to +4 of intron 6, affecting the canonical donor splice site (delG+1T+2G+3A+4). As a consequence of this rearrangement, the consensus GT is preserved at positions +1 and +2, whereas an altered motif (G+1T+2C+3T+4) is created at positions +3 and +4. Our in vitro analysis confirmed a deleterious effect of c.940+3_940+6del on pre‑mRNA splicing, with two aberrant transcripts identified. In one transcript, six nucleotides of LDLR intron 6 were retained; in silico analysis predicts a frameshift and a premature termination codon at amino acid position 371.

In the second transcript, the entire intron 6 of LDLR together with the intron of pET01 were retained in the mRNA, which is likely to result in the synthesis of a protein with an altered amino acid sequence or in mRNA degradation. Additional studies at the protein level will be required to confirm these predicted consequences.

Similar intronic donor-site variants in LDLR have been reported previously. The intronic variant c.1358+3_1358+8del, which deletes six nucleotides at positions +1…+6 while preserving GT at +1 and +2, has been described in a proband with an FH phenotype. Although functional studies have not been performed, segregation data suggest that this variant is likely pathogenic (Marduel et al., 2010).

Variants located at intronic position +5 are frequently classified as VUS in routine analysis. However, the +5 position of the donor site participates in binding to the 5ʹ end of U1 snRNA and is one of the determinants of donor site strength; substitutions at this position can reduce spliceosomal recognition efficiency (Malard et al., 2022).

Several LDLR variants at intronic position +5 have previously been investigated. The c.1186+5G>A variant (intron 8) leads to loss of the donor splice site and inclusion of intron 8 in the mRNA, followed by nonsense‑mediated mRNA decay (Etxebarria et al., 2012). In contrast, for c.1586+5G>A and c.2140+5G>A, only small amounts of mutant transcripts and no significant effect on protein activity were observed (Bourbon et al., 2009; Holla et al., 2009). In our study, substitution of G by A at position c.2389+5 resulted in exon 16 skipping from the transcript, and subsequent in silico analysis predicted loss of amino acid residues 771–796 and production of a truncated LDLR protein.

The acceptor splice site is characterized by highly conserved nucleotides at positions −2 and −1, which are recognized by the U2AF35 subunit, while the larger U2AF65 subunit binds to the upstream polypyrimidine tract. Variable positions near the AG dinucleotide, including −3, can modulate site strength by influencing U2AF heterodimer binding affinity to pre‑mRNA (Jenkins et al., 2013; Voith von Voithenberg et al., 2016).

Although intronic position −3 is not itself conserved, its functional relevance has been demonstrated experimentally: mutations at −3 account for ~4 % of all reported acceptor-site defects in inherited disease genes and lead to activation of cryptic splice sites or intron retention in vivo (Vorechovský, 2006). In our study, the c.941-3C>G variant caused loss of the canonical acceptor site and inclusion of part of intron 6. Substitution of cytosine by guanine at −3 disrupts the pyrimidinerich composition typical of the region adjacent to the acceptor AG dinucleotide.

Because U2AF65 recognizes the polypyrimidine tract upstream of AG, introduction of a purine in close proximity to the site likely weakens U2AF binding and promotes activation of a cryptic splice site. Depending on the surrounding nucleotide context, different splicing abnormalities may occur. For example, c.2312-3C>A in intron 15 has been shown to disrupt the 3′ acceptor site and cause skipping of exon 16 in mature mRNA in patients with FH (Liguori et al., 2001), whereas c.818-3C>G in intron 5 leads to inclusion of two intronic nucleotides in the mRNA, with a presumed premature stop codon at amino acid position 274 (Benito-Vicente et al., 2015). Our data are consistent with these observations.

According to R. Chong et al. (2019), among variants with a deleterious effect on splicing (3.8 % of all analyzed substitutions in the ExAC database), 83 % are located outside canonical ±1.2 positions and are evenly distributed across exons and introns. Ø.L. Holla et al. (2009) reported that functional analysis of 18 intronic variants demonstrated significant splicing effects for 14 of them (78 %). Together with our LDLR findings, these data emphasize that variants beyond canonical splice-site dinucleotides are often functionally significant but tend to remain classified as VUS in the absence of in vitro confirmation.

Functional in vitro assessment of variant effects on splicing is therefore a necessary, though not always sufficient, condition for reclassifying a VUS to likely pathogenic or likely benign. As shown in the present study, experimentally confirmed aberrant splicing strengthens the evidence for pathogenicity; however, final classification is determined by the aggregate ACMG/AMP criteria as applied within the gene‑specific FH VCEP ClinGen framework (Chora et al., 2022). Thus, minigene assays represent an essential tool for verifying the pathogenicity of intronic LDLR variants located outside canonical splice-site dinucleotides, including for establishing a molecular diagnosis in patients with a clinical FH phenotype (Blakes et al., 2022).

## Conclusion

In the present study, all three intronic LDLR variants – c.940+3_940+6del, c.941-3C>G, and c.2389+5G>A – were shown to exert a pronounced deleterious effect on pre‑mRNA splicing in the minigene system, leading to loss of canonical splice sites, activation of cryptic sites, and formation of aberrant transcripts. These findings underscore the need for functional analysis to substantiate the pathogenicity of variants for which in silico splicing predictions are inconclusive or discordant.

## Conflict of interest

The authors declare no conflict of interest.
